# Hidden family rules: perspective on a dysfunctional paternalistic system and the persistence of pain

**DOI:** 10.3389/fpain.2023.1303853

**Published:** 2023-12-14

**Authors:** Matt Hudson, Mark I. Johnson

**Affiliations:** ^1^ Centre for Pain Research, School of Health, Leeds Beckett University, Leeds, United Kingdom; ^2^ Mind Help Limited, Durham, United Kingdom

**Keywords:** family, family rule, pain, folie à deux, dysfunctional paternalistic system, painogenicity, emotional memory image (EMI), Split-Second Unlearning

## Abstract

This article explores how paternalistic control and power reside within the family system and how this may influence pain and its persistence. Drawing upon clinical case studies and existing literature, this exploration emphasises the role of paternal dysfunction in creating emotional memory images and delves into how this may influence the chronification and treatment resistance of pain (i.e., making pain “sticky”). We argue that a dysfunctional paternalistic family system, often characterised by authoritarian dynamics, emotional neglect, and abuse, results in adverse experiences and emotional memory images that create a fertile ground for the entrenchment and propagation of psychosomatic symptoms, including pain. Further, the paper emphasizes the potential intergenerational effects of such a scenario, where inherited “Family Rules” drive maladaptive coping mechanisms, which contribute to the persistence of psychological and physiological distress across generations. Understanding these complexities offers new perspectives on treating psychological disorders and their physiological ramifications. It also highlights the urgency of addressing dysfunctional familial dynamics in psychotherapeutic interventions for both immediate and long-term psychophysiological health outcomes.

## Introduction

A nurtured child forms the foundation for health, wellbeing, and human development (1). Family strain and dysfunction have a negative impact on the flourishing of a child (2). Childhood adversity is associated with poorer health and the occurrence, severity, and impact of chronic pain in later life (3–9). Chronic pain negatively impacts family dynamics (10–12), and conversely, family strain negatively impacts pain experience (13–16). Chronic pain in parents is associated with non-specific chronic pain in adolescents and young adults (3, 17–21).

Paternalistic system power remains on the periphery of a pain practitioner's clinical practice. Adversity and trauma resulting from paternalistic system power may influence chronification and treatment resistance of pain later in life (i.e., making pain “sticky”). Pain stickiness is a nickname introduced by Borsook et al. (22) to capture numerous social, psychological, and biological factors that influence pain persistence, pain behavior, and resistance to therapeutic intervention. Borsook et al. argued a need for neurobiologically informed psychotherapy, focused on pain as a motivational drive to avoid harm, to assist people in overcoming pain behavior that prevents exploration of possible alternatives to a life with persistent pain. Our perspective is that “Family Rules” causing adversity and trauma produce emotional memory images (EMIs) that may contribute to pain stickiness.

Reviews of the literature provide evidence that internal family systems contribute to chronic pain and treatment outcomes and should be a target for intervention (23–25). In 2023, a systematic review by Nicolson et al. of 68 studies found strong associations between chronic pain in adulthood and a broad range of adverse childhood events including household dysfunction, bullying, living in fear, emotional trauma, and weak parental attachment (9). Nicolson et al. (9) identified 15 different types of adverse childhood experiences, with emotional abuse being common, although there were no studies that specifically investigated childhood adversity through the lens of paternalistic family system power. In this article, we seek to uncover where paternalistic control and power reside within the family system, and how this may influence pain and its persistence.

## Family rules, myths, and secrets

Within an open paternalistic system of a family dynamic model/framework, shared responsibility among parents/caregivers allows the child to freely express their fears, hopes, and dreams (26). A closed paternalistic system demands power and control over those within it, creating censorship, isolation, and fear (27, 28). Satir suggested that family members would develop various behavioural strategies termed “Survival Stances” to describe how individuals acted out their roles to “survive,” such as placating, blaming, super-reasonable, and irrelevant (27).

The Family Rule maintains a closed paternalistic system. When a parent (or caregiver) is perceived as uncaring by the child or is caught doing something that they should not be doing, the parent may attempt to suppress the child’s knowledge of the event by explaining that the child has misunderstood the situation. By discrediting the child's understanding of a situation in this way, a parent “saves face,” hiding the event from others and psychologically suppressing the event for the child and the parent. As the child grows older, the experience may be assigned to a myth or kept a secret, following a Family Rule of maintaining silence out of fear of reprisal. It is as if the secret “hangs” in the relationship as an “ever-present noose,” ready to tighten if it should ever be approached (29).

Societal norms that prioritize materialism through a culture of having rather than being contribute to the decline of mental and physical wellbeing, as the focus becomes possessing rather than experiencing (30, 31). When fundamental human needs for autonomy, relationships, security, and purpose are not met, the detrimental effects of materialism become more pronounced (32). Consequently, individuals conditioned to adhere to these norms may favor materialistic remedies such as medication over conversational therapies (33, 34). Thus, we posit a pain patient raised in a closed paternalistic system perpetuating harm and dysfunction via a Family Rule would favor biomedical physicalist or mechanistic explanations for their pain and would seek “physical treatments” for a “physical ailment,” even when they are aware that they have been a casualty of psychological trauma. Speaking out or asking for help from the clinician may be psychologically impossible for the patient, as they may be trapped inside a subconscious Family Rule, which enforces the rule of the dominant family member over the health and wellbeing of the individual. The patient may aggressively or passive-aggressively deny any psychological sensitivity. In contrast, a pain patient raised in an open paternalistic system may present to the clinic able to discuss any emotional factors that may be contributing to their pain.

## Family system power—a metaphorical folie à deux?

Here, we use the concept of a folie à deux as a metaphor to enlighten the pain practitioner about the nature of patients who may have a non-conscious Family Rule. Folie à deux is considered a delusional belief system held by two or more individuals within the field of mental illness. In 1860, Jules Baillarger coined the term “folie à communiqué” to describe a shared psychotic disorder, and in 1877, Laségue and Falret coined the phrase “folie à deux” (“madness for two”) to describe a psychotic disorder shared by two people. American psychiatrist Theodore Lidz and his colleagues carefully observed 14 families of people diagnosed with schizophrenia (35–37) and found that behaviors would be shared between the dominant individual (inducer) who would apply rules to the family (induced) (38, 39) but not to themselves, which to the “induced” would appear as an admonition—“Do as I say, not as I do.” The inducer would clearly set themselves up to be seen as a hypocrite, yet the rest of the induced family would suffer punishment if they were to point out that this was the case—“the Emperor has no clothes” (40, 41).

Originally, it was assumed that the primary partner (the inducer) who initiated the delusions had a domineering personality, and the secondary partner (the induced) was generally submissive. This idea has been superseded by the concept of an adaptive mutual delusional system that allows members and partners to identify with each other, channel aggressive drives, and preserve intimacy (42–45). Today, the term folie à deux has been broadened to encompass a delusional belief system concurrently held by two or more individuals, so despite the wording, the disorder is not confined to pairs; it can encompass larger groups, adjusting to “folie à trois,” “folie à famille,” (44) and “folie en société” (46). This is to represent the number of people engulfed in the shared delusion. In psychiatry, a folie à deux is analogous to the “double bind” first proposed by Bateson in 1956 (47), as a miscommunication within a paternalistic relationship that can lead an individual to schizophrenia.

We caution that our use of folie à deux as a metaphor should not be interpreted to imply that pain is a form of psychosis. Our viewpoint is that the Family Rule is a form of miscommunication within a closed paternalistic system, a metaphorical folie à deux and double bind, that is a potent source of EMIs that, in turn, are realized as changes in neurobiology, including neurophysiological sensitization and bioplasticity that contribute to hyperalgesia, allodynia, and “sticky pain” (48). Furthermore, the relationship between cognitive distortions induced by dysfunctional closed paternalistic systems and pain is complex and far wider in scope than simply a shared delusional disorder (29), e.g., to include family system effects on assuming a “sick role” and illness behaviour (49).

## Family rules and emotional memory images

Emotional memory images are central to our theory of Split-Second Unlearning and psychophysiological disease. We proposed that adverse, emotionally overwhelming first-time experiences create EMIs (50) defined as “Trauma induced, non-conscious, contiguously formed multimodal mental imagery, which triggers an amnesic, anachronistic, stress response within a split-second” (51). In brief, traumatic events instigate psychophysiological stress responses and the formation of EMIs within very short “split-second” time frames, and these EMIs can be retriggered in daily living “replaying” stress responses, the recurring nature of which results in chronic “disease.” We posit that a Family Rule creates a multimodal EMI resulting from auditory (verbal and non-verbal sounds) and visual cues from the inducer, such as anger, disapproval, and rejection.

The EMI “holds” the unspoken, non-conscious rule, which must be obeyed to prevent severe punishment. The ultimate punishment as an infant may be abandonment by the parent or caregiver (inducer) or the withholding of love and attention (29). Generally, adults can rationalise rejection, whereas cognitive areas of the brain are still developing in children, and therefore, a “primitive” fight–flight–freeze reaction applicable to survival is more likely, i.e., the sense that the situation is “life or death.” In infancy and childhood, fighting or fleeing are implausible options, so freeze responses of hyperarousal or hypoarousal are activated to avoid punishment. Bateson et al. (47) suggest that repeated experiences embed adversity, although we assert that EMIs are a contiguously formed response to a single first-time emotionally overwhelming experience.

The EMI is formed when a child is in a state of heightened vulnerability and is learning to grapple with ambiguity and uncertainty stemming from the parent or caregiver's actions. This “learning” becomes encoded within the EMI. Empirical research into adverse childhood experiences underscores the profound psychophysiological repercussions in adults who endured harm during their formative years (52–55). Hence, the EMI traps the child in a psychological double bind, in which they seek assurance from a figure whose behavior is inconsistent. This predicament may precipitate an intolerance of uncertainty, compelling the individual to eschew scenarios that could trigger the EMI [see (56, 57)].

Activation of a Family Rule results in a non-conscious limbic system sympathetically mediated freeze response. In stressful circumstances that enable fight or flight, stress hormones are produced to mediate energy-consuming physical activity. The freeze response, however, puts the person into a heightened state of “pause,” and the person is held in a perpetual state of hypoarousal, manifesting as withdrawal and avoidance of sensory stimuli. This may explain, at least in part, the sluggishness observed in psychophysiological states of disease including depression, chronic fatigue syndrome (myalgic encephalomyelitis), and chronic primary pain (58). Chronic primary pain is defined as pain associated with significant emotional distress or functional disability that is not better explained by another chronic pain condition and includes non-specific chronic musculoskeletal pains (e.g., low back pain, neck pain), widespread pain, fibromyalgia, and irritable bowel syndrome (58–60).

The Imbalance of Threat and Soothing Systems model, proposed by Pinto et al. (61), corroborates this theory. In the Family Rule scenario, the freeze response is an optimal survival strategy as the induced do not have the fortitude to flee from or fight the perpetrator. Thus, children or vulnerable adults remain subservient. Releasing a person from a state of hypoarousal may enable a person to act out a fight or flight, assisting escape from the freeze response. We posit that the censorship created by the inducer proliferates the delusion, and the threat of neglect or abuse creates an EMI in the “induced,” suppressing their “spirit,” resulting in their silence.

We contend that EMIs facilitate psychophysiological disease through dysregulation of the hypothalamic–pituitary–adrenal (HPA) axis, triggering the stress response that augments physiological processes associated with persistent (primary) pain. Genes influence the response of the HPA axis to traumatic events in early life (e.g., FKBP5 and CRHR1 polymorphisms) (62) and how people respond to experiences in early life (63) and in adulthood (64); this may affect risk for chronification of pain (65). Borsook et al. (22) focused their discussion of the stickiness of pain on the contribution of neurobiological processes to a “stuck pain state”, including stress-induced epigenetic modifications, central sensitization, synaptic plasticity, HPA axis activity, brain circuitry, and opioidergic and dopaminergic tone, and how these may influence vulnerability or resilience to chronification. We advocate exploring the relationship between paternalistic family dynamics and the stickiness of pain at neurobiological and psychological levels, including health anxiety and reinforcement through caregiving behavior by formerly hostile or critical family members.

Previously, we have described a Split-Second Unlearning theory as a therapeutic framework to diagnose and “clear” EMIs created by trauma and adversity (50). Here, we describe how Split-Second Unlearning techniques can be used for clients presenting with trauma and adversity arising from codes of conduct (behavioral rules) imposed on family members by a dominant family member who does not follow the behavioral rules themselves.

## Childhood abuse and trauma

There is strong evidence that adverse childhood experiences are associated with mental health disorders and persistent pain later in life (5, 54, 66, 67). Adverse childhood experiences include emotional and physical neglect and sexual, physical, and emotional abuse. Adverse childhood experiences that are created during a child's formative years when the brain is developing, may leave a lasting imprint within the brain structure or, at the very least, an EMI that perpetuates a cascade of molecular and neurobiological effects, which hinders neuronal development (68, 69). This past adversity subsequently becomes the lens through which the child filters their experience of the world around them. This “experience” can develop into psychiatric symptoms like psychosis, aggression, or anxiety, as well as hostile behaviour as non-conscious physiological processes in the brain “hide” the person's awareness (observer) from the perpetual threat (70).

Globally, it has been estimated that up to 1 billion children have suffered abuse within dysfunctional Family Rule structures (71–74), contributing to adversity and the potential for intractable pain later in life (3–5). Previously, we have explored “Past Adversity Influencing Now” (PAIN) through the lens of temporal language and how this may impact the persistence of pain (75). Considering the relationship between hidden Family Rules and PAIN can assist the practitioner in affecting a positive outcome for the patient presenting with persistent pain.

## Detecting PAIN in clinical practice

The literature on childhood and preverbal trauma suggests that adverse experiences associated with Family Rules are difficult to identify. This is likely due to the young age at which adversity has taken place, within the child's development, and the nature of the traumatic event, e.g., varying severity of sexual, physical, or psychological abuse.

In 2023, we introduced a framework called PAIN to encourage exploration of pain through a temporal lens, guiding individuals toward a more positive future (75). Our PAIN framework encompassed temporal phases of pain: past perfect, past imperfect, present, future imperfect, and future perfect. We suggested that EMIs may contribute to a future imperfect and “sticky pain.” We described how detecting PAIN requires the practitioner to observe the client's eyes, breathing, voice tonality, and skin tone while completing a case history. The client may not be able to answer obvious questions such as “Were you raped or severely traumatized as a child?” due to the amnesic and anachronistic nature of the EMI formed by the original trauma (51), serving to “keep the family secret safe.”

**Figure 1:**
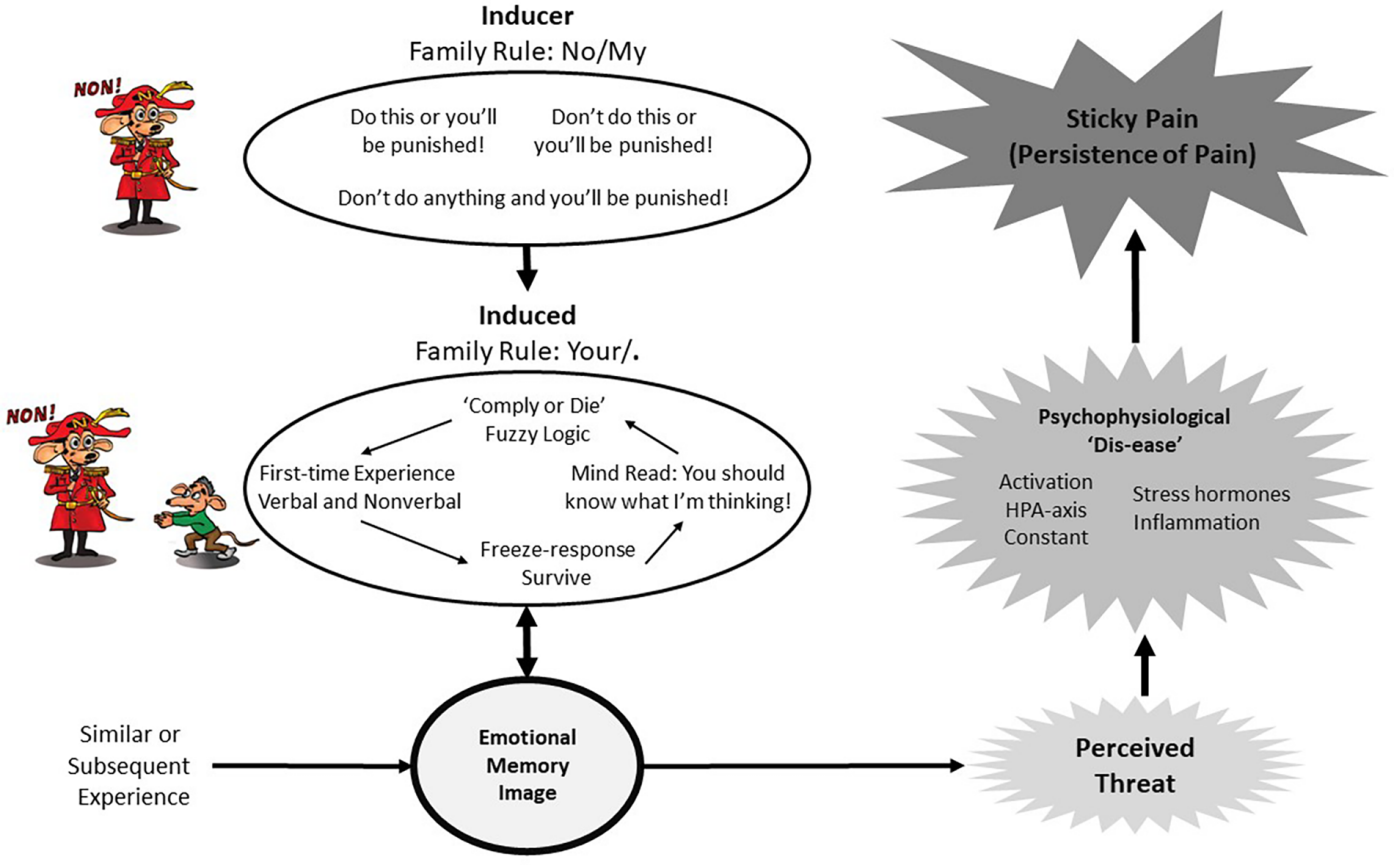
Family rule process.

The practitioner can gently coax the client by explaining that EMIs are created via experiences that are emotionally overwhelming at the time. For example, a parent or caregiver yelling at a child playing with a spider, “Don't touch the spider!” produces a fear-induced EMI in the child. Prior to the yell, the child was not afraid of the spider, and the new EMI motivates fear-avoidance reactions to spiders, promoting safety and survival in future encounters. EMIs are created within a dysfunctional paternalistic system, whereby as a child the individual is powerless and complies with the wishes of the “ruler” (inducer), even when illogical, to “survive.” This makes sense to those who are within the paternalistic system but may appear to be “fuzzy logic” to an outsider (see Figure 1). By providing the client with a simple explanation of this process, practitioners “allow” clients more scope to be open to the prospect of having PAIN.

## Structure of compliance

Children are reliant on parents or caregivers as a route to survival. From an evolutionary perspective, children are adapted to promote behaviors that seek alliances with parents or caregivers who provide protection and access to food. Disobeying parents or caregivers may be catastrophic to health and wellbeing, with the possibility of death. Parents or caregivers may impose codes of conduct (behavioral rules) on their children without following the behavioral rules themselves. Neurolinguistic programming (NLP) describes this process from the perspective of the parent or caregiver as a No/My rule structure, i.e., No rules for me/My rules for you. Compliance is the “safest” strategy for a child to “survive” (exist without confrontation) in this rule structure; from the child's perspective (induced), the rule structure is Your/. (period), i.e., your rules for me, full stop—there are no other rules (76). A synopsis of Family Rule structures is provided in Table 1, where the EMI is seen as a psychophysiological heuristic.

**Table 1:** Rule structure metaprograms.

Family Rule	Description	Example
1. My/My 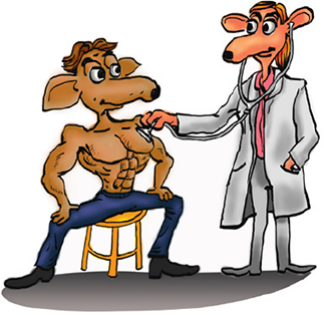 These individuals have rules for themselves, and they believe others should have the same.	My rules for me, my rules for you “If you want it to work then follow my instructions.”	“I’ve learned to live with my pain, you must learn to live with your pain too!”
2. My/. (period) 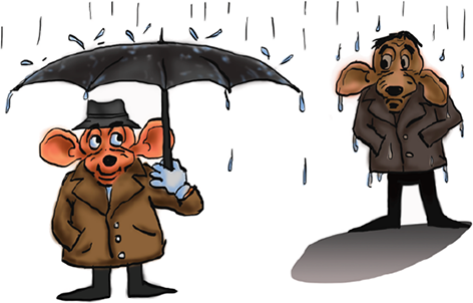 The individual is not concerned about others, seeking only what concerns themselves—period!	My rules for me, full stop (i.e., there are no other rules) “I did it my way.” My/. is synonymous with My/No—My rules for me/No rules for you—I’m not bothered about your rules.	“I’m not bothered about statistics I am talking about my pain”
3. My/Your 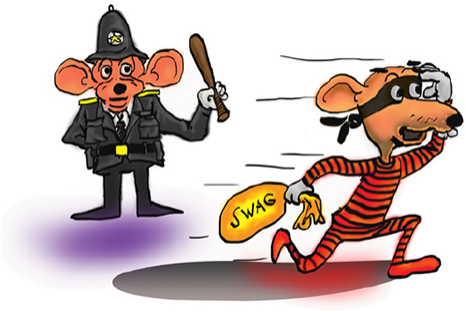 These individuals have rules for themselves but do not impose them on others.	My rules for me, your rules for you “Each to their own.”	“Better lifestyle choices cleared my pain, but you’ve got to find what's right for you”
4. No/My 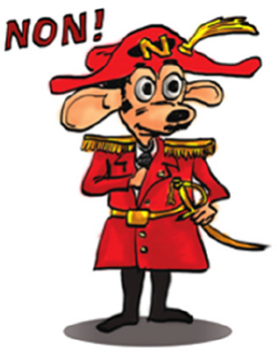 These individuals have no rules for themselves and apply rules to others.	No rules for me, my rules for you “Do as I say not as I do.” No/My is synonymous with Your/Your—Your rules for me (I must obey you), your rules for you (you can do what you want)	“I have no idea how to clear my pain, but I know what you should do to clear yours”
5. Your/. (period) 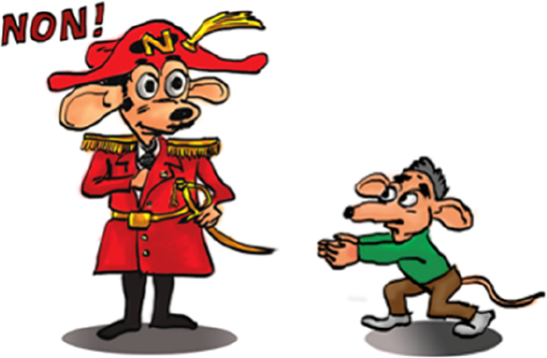 These individuals are oppressed and will comply with whatever their oppressor decides.	Your rules for me, full stop (i.e., there are no other rules) “Anything you say or do is alright with me”	“Whatever you want me to do to clear this pain I’ll do it”

Metaprograms act as cognitive shortcuts guiding an individual's decisions, behaviors, actions, and interactions. They represent the internal interpretation of the external reality of experience. These mental frameworks dictate both the focus of an individual's attention and the way they pay attention to things. In the table, rules 1–4 are identified within the NLP literature (see Charvet (78) for more detail on metaprograms). Rule 5 is a new rule created by Hudson (76) that allows for compliance and oppression, leading to “dis-ease” and must be present for an individual to be compliant and survive in a dysfunctional paternalistic system.

Practitioners can incorporate knowledge about Family Rules when using Split-Second Unlearning techniques to “clear” an EMI from a client presenting with bodily pain with no ostensible pathology (e.g., chronic primary pain). PAIN can be revealed via microexpressions that manifest while taking the case history. Making the client aware of their microexpressions (i.e., a biobehavioral marker) indicative of an EMI and engaging the client in a curious exploration of PAIN may be enough to shift their conceptual understanding of their painful condition, opening new opportunities for recovery [further details on these techniques can be found in our previous studies (75, 77)].

## Case vignettes

Here, MH uses case vignettes as examples of Split-Second Unlearning to treat PAIN in practice.

### Case vignette 1

A 12-year-old girl presented with chronic, widespread, non-specific musculoskeletal pain. The girl had been adopted from an unkempt orphanage in Eastern Europe, where she had been placed by her birth mother. The girl's adoptive mother presented with anxiety regarding her daughter's adoption, and I believed that this anxious tension was translating to her adopted daughter, precipitating musculoskeletal pain. The adoptive mother was diagnosed as having an EMI of her daughter as an infant, resulting in PAIN. As the adoptive mother spoke, both the mother and the daughter sat with their eyes transfixed on the spot within the peripersonal space between them. The mother was encouraged to look through the image of the past and to see her daughter with today's eyes. This action simultaneously cleared the EMI and the Family Rule of compliance; without the EMI, no rule is necessary. Clearing the adoptive mother's EMI reduced all pain from the girl within 30 minutes of treatment. The client was given an appointment for an online follow-up call 1 month later, at which the child reported no recurrence of symptoms; this was maintained at the 2-month, 6-month, and 12-month follow-ups. It is as though the child's pain was acting as a cry for help on behalf of both the mother and child, who were trapped in PAIN.

### Case vignette 2

A 15-year-old girl with myalgic encephalomyelitis and chronic widespread non-specific musculoskeletal pain presented to the clinic with her mother. The girl had physically matured early, which her mother reported to be of great concern to her father. The mother reported that the father was lavishing extra attention on the girl to ensure his daughter did not start dating, destabilising the family system. It was suggested to the mother that this may be precipitating her daughter's symptoms while the daughter listened indignantly. It was fascinating to note the mother's eyes were averted, while her daughter's eyes were fixated. Both aversion and fixation are signs of fear. The mother was listening, avoiding looking at her daughter, and her daughter was glaring when the Family Rule was brought to their conscious awareness. This is against the rules! The EMI in this situation is held in place by the Family Rule “Though must obey!”; both are induced, and the EMI oppresses them. The action of naming the source frees all from the rule, including the father, and restabilises the family system. The client was given an appointment for an online follow-up call in 1-month. The mother reported that she had discussed our session with her husband, and the couple had begun spending more time on their relationship. Thus, the father had reduced the overwhelming attention given to his daughter. The daughter's pain and fatigue had diminished greatly. At the 6- and 12-month follow-ups, the daughter reported that she was without pain or fatigue.

### Case vignette 3

A 48-year-old woman presented with finger joint pain associated with rheumatoid arthritis that had persisted for the past 3 years. When talking about her past experiences, the client's eyes looked to her left; this was interpreted as an experiential timeline from left (her past) to the right (her future). Interestingly, when the client spoke of her arthritis, her eyes fixated upward and to the right. This was interpreted as indicative of an EMI that was generalizing over time. I described my observations and deductions to the client, who appeared astonished and began to recall a conversation with her mother approximately 35 years earlier. The client explained that her mother had early-onset rheumatoid arthritis at age 45 and had been told that if she had a daughter, the daughter would suffer the same fate. This EMI, created from the conversation with her mother, remained dormant until the client reached 45 years of age. As the client spoke, she displayed a PAIN; her eyes were wide as she appeared detached from the present and fully associated with her EMI of the past. By bringing the EMI to the client's awareness and explaining how her mother's adversity had transferred to her, the EMI cleared, and the client was fully associated with the present once more. At the 1-month follow-up appointment, pain and swelling had diminished, and flexibility had returned to the client's fingers. At the 6- and 12-month follow-ups, there was no recurrence of any pain.

### Summary of case vignettes

The case vignettes demonstrate that bodily pain may be driven by PAIN. Attention to PAIN within a framework of Split-Second Unlearning may rapidly resolve bodily pain by clearing an EMI, allowing the HPA axis to return to a prestress state. The vignettes are examples of psychotherapeutic intervention; however, practitioners must appreciate that these clients did not require “talking therapy.” Often, clients are unaware that they are traumatised, either with a capital T or a lowercase t, although they are aware of bodily pain and require help from a practitioner. In vignettes 1 and 2, the parent and child must be present for the Family Rule to be broken or cleared. By gaining new insight into the psychological aspect of pathology, no blame is apportioned to the caregiver, and the EMI is cleared. We concur with Ecker and Vaz (79) that the process of erasure clears any psychophysiological attachment to this emotional learning. In all cases, the Family Rule is the elephant in the room, which will continue to create misery and pain if the practitioner is unable or unwilling to address it.

## Closed paternalistic systems and painogenic environments

In a broader context, closed paternalistic systems with the potential for dysfunctional rules, structures, and maladaptive beliefs can operate at various levels in society. This may include idiosyncratic beliefs of an individual, beliefs shared by a few individuals (shared delusions), and beliefs shared by subgroups, subcultures within and between communities, regions, and nations, resulting in, for example, prejudice, discrimination, or dogma. A “collapsing tin can” metaphor describes people living within a “closed-societal system”: A social milieu of threat, fear, and anxiety mediated by complex societal structures, settings, and narratives compresses the mental wellbeing of individuals, creating a closed societal system, like high atmospheric pressure compressing the walls of a tin can with a low-pressure interior. Forces producing this constraining milieu may be insidious and invisible to individuals (32, 80, 81). Individuals utilize a variety of strategies for existing and surviving within the pressure of the closed system. Psychophysiological disease may be a consequence and/or a survival strategy of being immersed in insidious macrolevel forces. This promotes the rise in mental illness and non-communicable diseases, including chronic primary pain, chronic fatigue syndrome, depression, generalised anxiety, and so on (82).

Societal settings creating closed systems operating within a No/My authoritarian rule structure (i.e., No rules for me/My rules for you) may fall foul of groupthink. Groupthink is when no one will challenge the thoughts of a group and people just go along blindly obeying in a Your rules for me/full stop fashion (i.e., Your/.).

“They are playing a game. They are playing at not playing a game. If I show them I see they are, I shall break the rules and they will punish me. I must play their game, of not seeing I see the game” (83, p. 1).

We contend that a dominant biomedical groupthink may be detrimental to alleviating the societal burden of chronic pain, leading to personal suffering. Insights may be gained by exploring dysfunctional paternalistic systems within the socio-ecological framework of painogenicity (84). The concept of painogenicity provides a socio-ecological framework to explore the persistence of pain (84, 85). Painogenicity is the sum of influences that the surroundings, opportunities, or conditions of life have on promoting persistent pain in individuals or populations, encompassing micro-, meso-, and macrolevel factors (85, 86). Macrolevel factors such as built or natural habitats, geopolitics, and economic sectors are often neglected in models of pain, despite their potential to foster a social milieu of threat, fear, and anxiety through illness narratives of pain grounded in tissue damage, pathological causation, and warmongering of biomedical remedy (80, 87).

Increasingly, scholars argue that biomedicalisation of mental health conditions and chronic (primary) pain has perpetuated rather than diminished the burden of disease (32, 88). No doubt, biomedical remedy assists resolution of episodes of pain in many people, some of the time, although the global burden of persistent pain remains high irrespective of a country's social and economic development and despite ever-increasing varieties of treatment (89–91). We advocate exploration of this treatment-prevalence paradox through the lens of a closed paternalistic biomedical system to deepen an understanding of socio-ecological factors influencing the persistence of pain and other intractable non-communicable diseases, including mental illness.

## Conclusion

In this article, we describe how dysfunctional family structures may lead to Family Rules resulting in PAIN and EMIs that contribute to the persistence (stickiness) of bodily pain. We describe a Split-Second Unlearning approach to “clear” EMIs and unblock the detrimental effects of PAIN, with the potential for a “healing journey” toward recovery from persistent pain (92). We position this approach within a salutogenic framework of care (93) that may be more successful than biomedical interventions in “unsticking” pain.

We conclude that exploring pain and its persistence within a dysfunctional paternalistic context could deepen an understanding of factors contributing to chronification and treatment resistance and may provide opportunities to assist people on a “healing journey”. Emphasizing a holistic, socio-ecological model of pain encourages healthcare practitioners to think beyond traditional diagnoses and treatment strategies. In this regard, consideration of the influence of family dynamics and psychosocial factors on a person's experience of persistent pain may improve intervention strategies and potentially break intergenerational cycles of disease.

## Data Availability

The original contributions presented in the study are included in the article/Supplementary Material, further inquiries can be directed to the corresponding author.
